# Maternal inheritance of primary sex ratios in the dark-winged fungus gnat *Lycoriella ingenua*

**DOI:** 10.1038/s41437-026-00821-0

**Published:** 2026-01-29

**Authors:** Maria Shlyakonova, Katy M. Monteith, Laura Ross, Robert B. Baird

**Affiliations:** 1 Institute of Ecology and Evolution, University of Edinburgh, Edinburgh, UK; 2 Present Address: Whitehead Institute for Biomedical Science and Howard Hughes Medical Institute, Cambridge, MA USA

**Keywords:** Evolution, Evolutionary genetics

## Abstract

Sex determination mechanisms in insects are extraordinarily diverse, although most species have zygotic genotypic sex determination where sex is established by sex chromosomes upon fertilisation. Dark-winged fungus gnats (Diptera: Sciaridae) are a large and speciose family of flies where sex determination is a result of an unusual interplay of zygotic, maternal, and environmental factors. This causes some species to produce broods that deviate considerably from the standard 1:1 sex ratio. An early study suggested that these primary sex ratios may be heritable from mother to daughter, but this observation has not been corroborated and the genetic basis for this trait remains unknown. Other studies have found that in some species, there is an additional temperature effect on the primary sex ratio, but again the mechanism is unknown. Here, we perform sibling crosses and temperature-shift experiments in the common mushroom pest *Lycoriella ingenua* and find evidence for highly variable and heritable primary sex ratios, but no significant environmental effect. We discuss the consequences of our findings for understanding the mechanisms that produce these unusual sex ratios, and the evolution of sex determination more broadly in this clade.

## Introduction

Sexual reproduction is an ancient feature in eukaryotes, yet the mechanisms by which offspring sex can be determined are strikingly diverse (Beukeboom and Perrin, 2014; Bachtrog et al., 2014), and this is particularly true of insects (Blackmon et al., 2017). Most sex determination systems are genotypic, where loci on the sex chromosomes determine offspring sex. For example, in the housefly *Musca domestica*, a Y-linked factor acts as the primary signal for sex determination (Hediger et al., 1998); in the fruit fly *Drosophila melanogaster*, it is the dosage of an X-linked transcript (Erickson and Quintero, 2007). There are, however, many exceptions to the rules. For example, genotypic sex determination does not always involve distinct sex chromosomes (Weber and Capel, 2021), a single master switch gene (Moore and Roberts, 2013), or even the evolution of separate sexes (Ghiselin, 1969). In some systems, sex is not genotypically determined, but rather environmentally (environmental sex determination, ESD), such as in many reptiles (Bull, 1980), fish (Godwin and Roberts, 2018), and some crustaceans (Kato et al., 2011). Such diversity demonstrates the dynamic nature of sex determination systems, which undergo frequent turnover in many clades (Vicoso, 2019). The sex determination mechanism directly influences the primary sex ratio: where sex determination is governed by X and Y chromosomes, the segregation of those chromosomes establishes the primary sex ratio (Werren and Beukeboom, 1998). Although sex ratio theory predicts that frequency-dependent selection should result in a 1:1 primary sex ratio, there are many exceptions, notably in Hymenoptera (wasps, bees and ants), where their haplodiploid sex determining systems allows females to facultatively control sex ratios. (King, 1987; Meunier et al., 2008). In other taxa, ESD can be a cause of unorthodox or highly variable sex ratios (Charnov and Bull, 1989; Korpelainen et al., 1997), although ESD is extremely rare in insects.

**Fig. 1:**
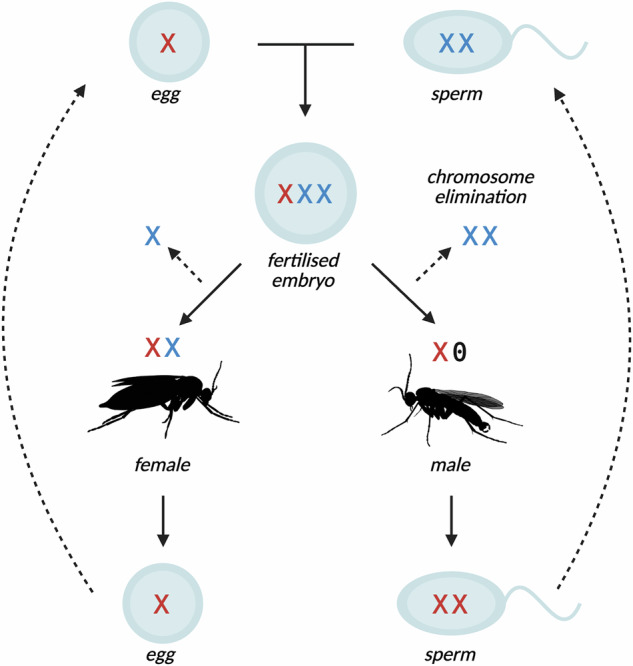
Simplified chromosome cycle of dark-winged fungus gnats (Sciaridae), showing inheritance of the X chromosomes. XXX zygotes form from the fusion of XX sperm and X eggs. Embryos that undergo elimination of one X chromosome develop into females (XX), those that lose two develop into males (X0). The first male meiotic division involves elimination of all paternally-inherited autosomes; the second division involves nondisjunction of the maternal X. As a result, males always produce sperm with two copies of the X.

The dark-winged fungus gnats comprise a family of flies (Diptera: Sciaridae) that seem to contradict virtually all perceived wisdom regarding chromosome inheritance and sex determination. Their unusual chromosome cycle was deciphered in the early 20th century through studies on various species that we now know to belong to the genera *Bradysia* and *Lycoriella* (Metz, 1938). Later studies on the genus *Trichosia* (Amabis et al., 1979; Fuge, 1999), which shares an ancestor with *Bradysia* at the root of the sciarid phylogeny (Shin et al., 2013), suggested that the cycle is probably a feature common to the whole family. Sciarids have a chromosome inheritance system called paternal genome elimination (PGE), a form of haplodiploidy where males inherit, but do not transmit, their paternally-inherited chromosomes (Metz, 1938). Whereas oogenesis is normal, every sperm produced by a male bears two copies of the maternally inherited X and one copy of the maternally-inherited autosomes. After fertilisation, all zygotes therefore have three X chromosomes. Sex is determined post-fertilisation in the early cleavage divisions, when either one or two X chromosomes, which are always derived from the sperm, are eliminated, giving rise to males (X0) or females (XX), respectively (Fig. 1). The elimination of X chromosomes occurs prior to zygotic genome activation (de Saint Phalle and Sullivan, 1996), so is presumably governed by factors that are maternally-deposited into the embryo.

The sex ratio of a brood, therefore, depends on how many embryos eliminate one or two X chromosomes. In some species, such as *Bradysia coprophila* or *Bradysia impatiens*, mothers produce broods of exclusively one sex (monogenic reproduction). These male-producers and female-producers are genotypically distinct, with the latter harbouring large inversion-based supergenes for which they are always heterozygous (Baird, Urban et al., 2023). Other species, such as *Bradysia ocellaris* or *Bradysia. reynoldsi*, produce mixed sex broods (digenic reproduction) (Metz, 1938). In digenic species, sex ratios are known to be highly variable. Single broods that are strongly sex-biased in both directions have been reported in *B. ocellaris* (Davidheiser, 1947; Nigro et al., 2007), *Bradysia matogrossensis* (Rocha and Perondini, 2000), and *Bradysia odoriphaga* (Cheng et al., 2017). Monogenic species are also known to produce occasional ‘exceptional’ offspring of the wrong sex (Metz and Schmuck, 1929), suggesting that the distinction between monogeny and digeny may be one of degree, rather than kind. Variance in primary sex ratios, as well as maternal effects on sex ratios, are thought to be indicators of polygenic control over sex determination (Bull, 1983). We have previously suggested that the observations of variable sex ratios in sciarids imply that the proportion of a female’s embryos that develop into males versus females may have an additive genetic component (Baird et al., 2023).

Interestingly, results from a previous study investigating *B. ocellaris* indicated that daughters produce similar sex ratios to their mothers when mated to male siblings (Davidheiser, 1947), suggesting that primary sex ratios may be heritable. However, these experiments have not yet been repeated in any other dark-winged fungus gnat species, and heritability of sex ratios in this clade is based on small sample sizes and has not been verified statistically. Previous studies have also found a temperature effect on primary sex ratios in the digenic species *B. ocellaris* (Nigro et al., 2007) and *Lycoriella auripila* (Farsani et al., 2013). The temperature-sensitive period was found to be during the late pupal stage when oogenesis is taking place (Berry, 1941) and is the result of conversion of one sex into another rather than sex-biased mortality (Nigro et al., 2007). However, the temperature effects appear to be species-specific, with an increase in temperature causing more female-production in *B. ocellaris*, and both high and low temperatures resulting in more males in *L. auripila*.

Dark-winged fungus gnats, therefore, offer an opportunity to study sex determination where there is a unique interplay of zygotic, maternal, and environmental factors (Sánchez, 2010). In the present study, we isolated a line of the digenic species *Lycoriella ingenua* and established isofemale lines over six generations to investigate the genetic and environmental components of primary sex ratio variation. Specifically, we aimed to quantify the extent of variation in primary sex ratios, test whether sex ratios show evidence of maternal inheritance, and determine whether temperature during development influences primary sex ratios in this species. Addressing these questions has implications for understanding sex determination in the Sciaridae clade and whether maternal control of sex in sciarids is consistent with a polygenic basis.

## Materials and methods

### Insect collection and husbandry

A laboratory culture of a sciarid species was established from a wild population collected from Mycobee Mushroom Farm in North Berwick, UK (56.0234° N, 2.7872° W), in February 2022. Several hundred flies were collected over a period of 4–5 weeks, as they emerged from a mushroom substrate bag, to establish a laboratory stock. After this, the stock was maintained in mass cultures (~20 vials with several individuals of each sex per vial) at 18 °C and 70% relative humidity in 28 mm × 95 mm polypropylene vials containing 2.2% Bacteriological agar. Larvae were fed a mixture of brewer’s yeast, mushroom powder, spinach powder, and ground straw every day 2–3 days until pupation, similar to the method used to rear sciarids in the genus *Bradysia* (Gerbi, 2024). The laboratory stock was maintained for ~10 generations (1 year) before isofemale lines were established for the experiments in this study. The genus was tentatively identified by inspecting clasper morphology (Broadley et al., 2018), and the species identity was confirmed with a BLASTn (Altschul et al., 1990) search of the cytochrome oxidase subunit 1 (COI) barcode sequence (Folmer et al., 1994) using WGS data generated for another study (Baird et al., 2025). The species was also confirmed as digenic by rearing isofemale lines for a generation and observing mixed sex, rather than single sex (monogenic) broods.

### Primary sex ratios in isofemale lines

Isofemale lines were established from mass cultures at random: one male and one female were placed inside a vial and left to mate. A total of 47 isofemale G0 lines were initially set up, of which 42 produced eggs that hatched and survived to produce F1 offspring. Following pupation, vials were checked daily for eclosing F1 adults. The primary sex ratios were recorded as the proportion of male offspring that eclosed from each vial.

**Fig. 2:**
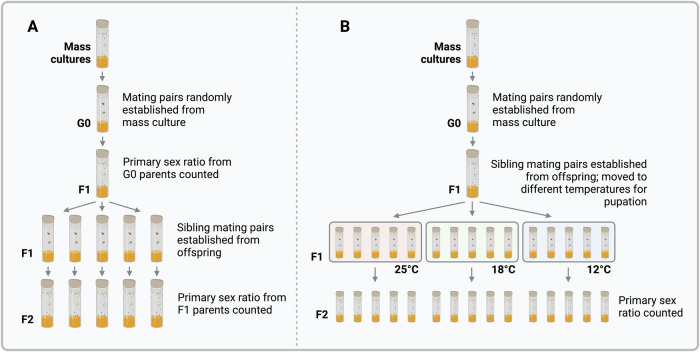
Schematic overview of experimental design in this study. **A** Experimental design to examine heritability of primary sex ratios. After establishing isofemale lines from mass cultures, sibling crosses were set up over successive generations and offspring counted; the last flies to be counted were the F4 generation (i.e. offspring of F3 females). **B** For the temperature experiment flies were either held at 18 °C or moved to 25 °C or 12 °C for the duration of pupation (oogenesis). Upon emergence, females that had been subjected to different temperatures for pupation were mated and their offspring counted.

In order to determine whether the primary sex ratios produced by mothers were heritable, sex ratios were also tracked through successive generations. Sibling crosses were established using eclosed F1 adults from 28 of the 42 successful lines, with the aim of performing 5 sibling crosses per clutch (i.e. 140 total crosses, although not all survived to the next generation). The above process was repeated to generate F2 primary sex ratio counts, and again for F3 and F4 counts, but setting up 3–5 sibling crosses per clutch, depending on the number of available offspring (Fig. 2A). Sciarid females eclose as adults with a complete set of mature eggs, only mate once, and usually die within hours of egg laying (Moses and Metz, 1928; Featherston et al., 2013). We did not specifically aim to collect virgins, because of the high likelihood that females should only mate once and were always mated with their siblings.

### Temperature-shift experiments

Some F1 offspring from the primary sex ratio experiment were used to perform temperature-shift experiments. A total of 15 F1 sibling crosses were established from each of the 28 lines (total of 420 crosses) and up to 5 crosses from each F1 clutch were randomly allocated to one of three different temperatures; 12 °C, 18 °C, and 25 °C. Previous studies report that temperatures above 27 °C are lethal to dark-winged fungus gnats, and that temperatures below 12 °C slow development by several months (Reynolds, 1938; Nigro et al., 2007). The temperature shifts were designed to encompass the pupal stage of the mother, when oogenesis occurs, which has previously been shown as the stage at which temperature affects the sex ratios (Nigro et al., 2007). This was achieved by moving the developing flies from 18 °C, the control temperature, to the shift temperature at the onset of pupation. The eclosing females were then mated with male siblings, and their offspring (the F2 generation) were reared at 18 °C and sex ratios were recorded upon eclosion (Fig. 2B).

### Statistical analyses

All data visualisation and analysis was done in RStudio (R Core Team, 2023). To test if primary sex ratios deviated from the 1:1 expectation, binomial tests were performed for each brood and *P* values were combined using Fisher’s method. To determine whether primary sex ratios were heritable, a linear regression was performed between the sex ratios produced by mothers and their daughters, weighting primary sex ratios by brood sizes to reduce noise from sex ratios based on small broods. This was done by scaling the primary sex ratio by the square root of the brood size separately for mothers and daughters. Whether siblings produced more similar primary sex ratios than non-siblings was assessed by calculating and weighting the absolute differences in sex ratios for siblings and non-siblings and performing a Welch two-sample *t* test on the weighted differences. To examine whether the variance in offspring sex ratio differed among isofemale lines, Levene’s test for homogeneity of variance was used, with founder line as a grouping factor and the median as the centre.

Changes in sex ratio biases across generations were examined by categorising broods as male-biased (>0.6), unbiased (0.4–0.6), and female-biased (<0.4), and fitting a multinomial logistic regression model with generation as a fixed effect and weighting each brood by the number of offspring. To see if brood size (i.e. offspring mortality) changed over generations, a mixed-effects model was used with brood size as the response, generation as a fixed effect, and founder line as a random effect. Pairwise contrasts were then conducted between successive generations. A generalised linear model was used to determine if the decline in brood size over generations was sex-biased, with brood size as the response variable, an interaction between generation and sex as a fixed effect, and founder line as a random effect.

To determine whether there was an effect of temperature on the sex ratio, a linear regression model was used, with primary sex ratios weighted by brood size. Changes in mortality in different temperature treatments were assessed using a linear model, and changes in sex-specific mortality using a linear model with offspring sex as an interaction term.

## Results

### Species identification

The population we collected from North Berwick, UK, was identified as belonging to the genus *Lycoriella* by its clasper morphology, and confirmed as *L. ingenua* (Dufour 1839) following barcoding using the COI sequence. Under the former designation *Sciara pauciseta* (Metz, 1938), *L. ingenua* has been described as digenic, which we confirmed by observing mixed sex broods after initial rearing of isofemale lines. At 18 °C and 70% relative humidity, we found that the life cycle of *L. ingenua* lasts ~4–5 weeks from egg laying to pupation.

### Variability and changes in primary sex ratios and survivorship

**Fig. 3:**
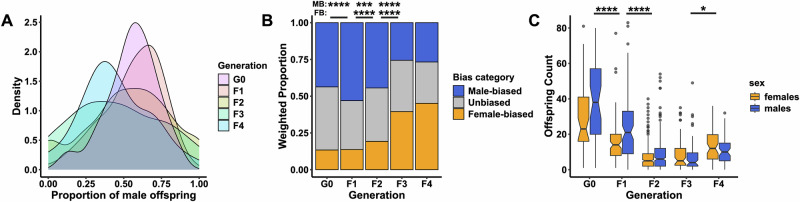
Sex ratio variance and survivorship. **A** Density plot showing variation in primary sex ratios for the five generations studied. **B** change in sex ratio bias over generations (weighted by brood size), with asterisks showing significant changes in the proportion of broods classified as male-biased (MB, >0.6) or female-biased (FB, <0.4) between successive generations, tested using a multinomial logistic regression with generation as a fixed effect. **C** Male and female offspring counts over generations, with asterisks showing statistical comparisons of clutch size between successive generations, tested using a linear mixed-effects model (brood size ~ generation + 1|founder line)). Asterisks represent significance levels (^****^P < 0.0001; ^***^P < 0.001; ^*^P < 0.05).

We found that sex ratios were significantly more variable than expected under a normal binomial distribution (Fisher’s combined *P* > 10^−135^). Although the mean and median proportion of male offspring across all progenies were 54% and 52%, respectively, primary sex ratios varied from 0% to 100% male offspring (Fig. 3A). Interestingly, between the founder generation (G0) and their offspring (F1), broods became significantly more male-biased (*P* < 0.0001), but between generations F1 and F3, they became significantly more female-biased (F1-F2 comparison: *P* < 0.001; F2-F3 comparison: *P* < 0.0001) and significantly less male-biased (both comparison: *P* < 0.001; Fig. 3B). We found that overall mortality increased over the first two generations (*P* < 0.0001), which could result from inbreeding depression in isofemale lines. As inbreeding could affect male and female fitness differently (Ebel and Phillips, 2016), these changes in primary sex ratios over generations could be due to sex differences in offspring mortality. However, we found that the extent of decline in offspring count over generations did significantly differ between males and females, suggesting this is not the case. We also found an increase in brood sizes between generations F3 and F4 (*P* < 0.05, Fig. 3C).

### Inheritance of the primary sex ratio

**Fig. 4:**
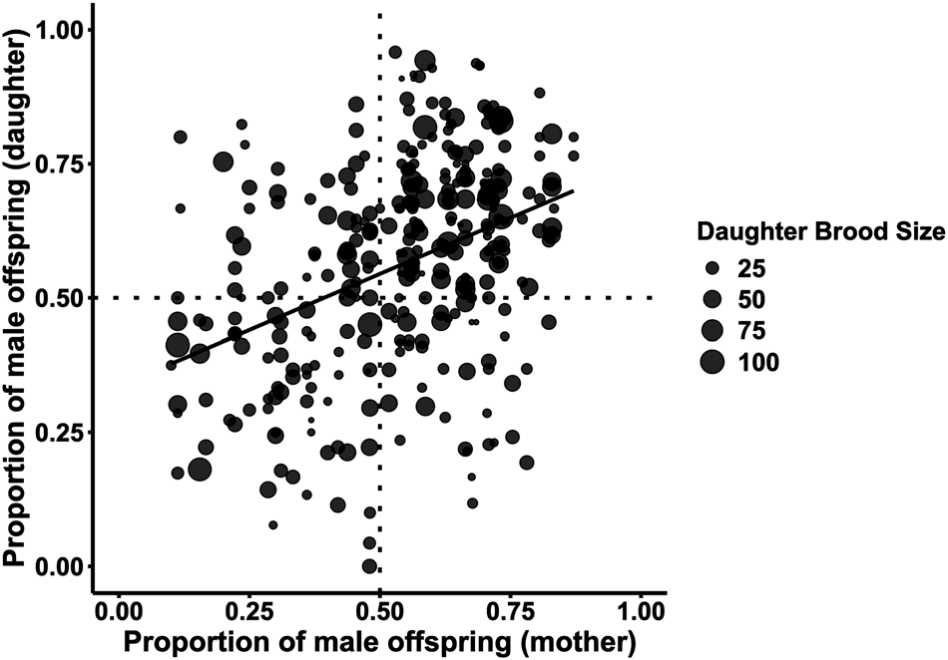
Mother versus daughter primary sex ratios. Each point represents the proportion of male offspring in a mother-daughter pair (only brood sizes >= 10 are shown). The regression line shows the fitted relationship from a linear regression (*β* = 0.27±0.04, *R*^2^ = 0.09, *P* < 10^−10^).

Females that were mated to their male siblings tended to produce similar primary sex ratios to their mothers. Specifically, a weighted linear regression revealed a modest but significant correlation between the primary sex ratio produced by a mother and that of her daughter (Pearson’s *r* = 0.30, *β* = 0.27±0.04, *R*^2^ = 0.09, *P* < 10^−10^). Although the effect size is small, the directionality of the relationship indicates that daughters of more male-biased mothers also tend to produce more male-biased broods, thus suggesting that primary sex ratios are heritable in *L. ingenua* (Fig. 4). In further support of this, primary sex ratios were significantly more similar between siblings compared to between non-siblings (Welch two-sample *t* = −9.5, *df* = 1892.5, *P* < 10^−15^). Moreover, daughter sex ratios showed significant heterogeneity in variance among isofemale lines (Levene’s test: F(27, 412) = 2.26, *P* = 0.0004), indicating that some lines consistently produced more variable sex ratios than others (Supplementary Fig. 1).

### Temperature effects

**Fig. 5:**
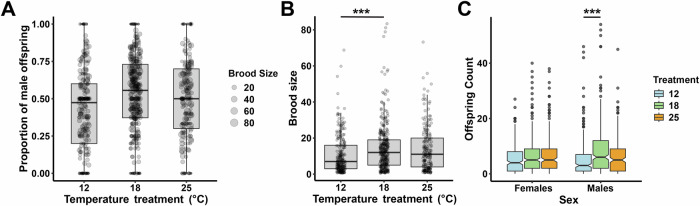
Temperature effects on development. **A** Effect of temperature on primary sex ratios, tested using a weighted linear regression. **B** Effect of temperature on brood size, tested using a linear model. **C** Effect of temperature on male and female counts, tested using a linear model with an interaction between sex and temperature. Asterisks represent significance levels (^****^P < 0.0001; ^***^P < 0.001; ^*^P < 0.05).

The median proportion of males produced by mothers reared at 12 °C, 18 °C, and 25 °C was 47%, 56%, and 50%, respectively. We did not find a significant effect of temperature on primary sex ratios (*P* = 0.971, Fig. 5A). We also tested for differences in mortality under the different temperature treatments, and found that offspring mortality was higher at both 12 °C and 25 °C compared to 18 °C, but the difference was only significant for the 12–18 °C comparison (Fig. 5B). There was no significant difference when considering only female offspring mortality, but there was for males at 12 °C, suggesting that males are perhaps more sensitive to changes in the temperature at which the mother was reared (Fig. 5C).

## Discussion

Dark-winged fungus gnats have an unusual system of sex determination where a combination of zygotic, maternal, and environmental factors influence the sex of offspring (Sánchez, 2010). Sperm with two X copies fertilise eggs with one X, producing XXX zygotes that proceed to lose either one or two X chromosomes in early embryogenesis, developing as XX females or X0 males, respectively. Whether a zygote develops as male or female can be influenced by the maternal genotype (Baird et al., 2023) and temperature during oogenesis (Nigro et al., 2007; Farsani et al., 2013). Because early embryonic development is under maternal control, the paternal genome is not expected to affect the sex ratio, although a male may influence the sex ratio produced by his daughters through alleles transmitted to them. In digenic sciarids, where females produce mixed sex broods, mothers are known to produce variable sex ratios (Rocha and Perondini, 2000; Nigro et al., 2007; Farsani et al., 2013), and results from a previous study suggest that the primary sex ratio may be heritable (Davidheiser, 1947). In the present study, we sought to determine the extent of variability, heritability, and the effect of temperature on primary sex ratios in a recently cultured laboratory strain of the species *L. ingenua* with the aim of improving our understanding of the genetic and environmental mechanisms that determine sex in this unusual clade of flies.

We found that primary sex ratios are highly variable in this species, ranging from 0% to 100% male offspring. This is concordant with observations from studies of other sciarid species, including *B. ocellaris* (Nigro et al., 2007), *B. odoriphaga* (Cheng et al., 2017), *B. matogrossensis* (Rocha and Perondini, 2000), *Lycoriella agraria* (Farsani et al., 2013), and *Scatopsciara cunicularis* (Sawangproh and Cronberg, 2016). That the primary sex ratio of a brood appears to be a continuous trait suggests that it is under the control of more than one locus, as suggested by Rocha and Perondini (2000) and expanded upon in Baird et al. (2023a). Our finding that primary sex ratios are more similar between siblings than between non-siblings, and that daughter sex ratios correlate significantly with mother sex ratios, provides a strong indication that the primary sex ratio has a heritable genetic component. Moreover, we also found significant heterogeneity of variance among isofemale lines, which is consistent with line-specific, heritable effects on sex ratio. However, there was considerable variation between siblings in primary sex ratios, suggesting that more than one locus or indeed environmental factors may be at play.

Some of the primary sex ratios we observed were highly skewed, and we found that sex ratios became increasingly female-biased over successive generations. This pattern did not appear to be driven by differences in male versus female mortality. We did, however, find that mortality increased over generations, which could result from inbreeding (although mortality decreased in the final generation, which could represent recovery after purging of genetic load and loss of lethal allele combinations, Crnokrak and Barrett 2002; Charlesworth and Willis 2009, Mongue et al. 2016). The trend towards female-bias over generations may reflect stochastic processes acting on sex ratio-influencing alleles following the strong bottlenecks imposed by establishment of the isofemale lines. For example, inbreeding may have fixed particular maternal-effect alleles that bias sex ratios toward the production of females.

Broods with similarly highly biased sex ratios are common in other digenic sciarid species (Davidheiser, 1947; Rocha and Perondini, 2000; Nigro et al., 2007). Some other sciarids, in contrast, produce strictly single-sex broods (Metz, 1938; Lara et al., 1965; Steffan, 1974), and in two cases this is known to be associated with large chromosomal inversions (Carson, 1946; Crouse, 1979). In Baird et al. (2023a), we proposed that these inversions evolve to ‘trap’ alleles that influence the sex ratio, resulting in a transition from digenic to monogenic reproduction, and that this may be a result of sex ratio selection. Documented cases of multi-locus or polygenic sex determination are rare and are thought to be only a transient phenomenon (Schartl et al., 2023; although see Kocher et al. 2024). Polygenic sex determination is thought to be inherently unstable (Rice, 1986; Bateman and Anholt, 2017), although some systems show remarkable stability. For example, the complex system of the housefly *M. domestica* has multiple male-determining alleles across different chromosomes and has persisted stably in natural populations (Feldmeyer et al., 2008; Kozielska et al., 2008; Hamm et al., 2015). Moreover, the potential instability of polygenic systems may also be exacerbated when environmental effects such as temperature influence offspring sex (Van Dooren and Leimar, 2003). In Sciaridae, the evolution of monogeny from digeny may be one way or resolving this instability.

In contrast to some previous studies on other sciarid species (Nigro et al., 2007; Farsani et al., 2013), we did not find a significant effect of temperature on primary sex ratios, suggesting that this temperature effect may be species-specific. Because the laboratory stock was maintained in relatively small mass cultures (3–4 adults of each sex per vial) after collection from the wild, it is possible that standing genetic variation present in the wild population was partially reduced before the establishment of the isofemale lines. As such, we cannot exclude the possibility that genetic variants affecting temperature sensitivity in sex determination may have been lost. Notably, however, the strain of *B. ocellaris* in which a temperature effect was found was maintained for ~40 years in laboratory conditions and was therefore probably highly inbred (Nigro et al., 2007).

The precise mechanism of temperature effects on sciarid sex determination is yet to be explored. Maternal factors that determine the sex of an embryo must be transferred from nurse cell cytoplasm to oocytes. Extreme temperatures may affect the rate of transfer, as suggested by Nigro et al. (2007) and Sánchez (2010), but to our knowledge the effect of temperature on maternal mRNA or protein deposition has not been studied. An alternative possibility is that extreme temperatures induce meiotic nondisjunction. Errors in female meiosis may result in eggs with aberrant karyotypes: because all eggs fuse with an XX sperm, nullisomic eggs would produce XX zygotes and disomic eggs XXXX zygotes. In typical systems, this would be fatal. However, under this system of sex determination, post-zygotic X elimination would restore some eggs to viable ploidy (e.g. if one X is eliminated from XX zygotes or if two Xs are eliminated from XXXX zygotes). Rearing *Drosophila melanogaster* at low or high temperatures has been known to induce meiotic nondisjunction (Grell, 1979), mostly affecting the X chromosome (Tokunaga, 1970). Furthermore, exceptional male and female offspring that occur in the closely related monogenic species *B. coprophila* can result from sex-chromosome nondisjunction during oogenesis, producing aneuploid eggs (Crouse, 1960).

## Conclusion

Inheritance of primary sex ratios is a rare phenomenon in nature because in most organisms sex is determined by Mendelian segregation of sex chromosomes and females produce standard 1:1 sex ratios. Here we demonstrate that in the dark-winged fungus gnat *L. ingenua*, in which sex is determined by maternally-controlled elimination of X chromosomes in the embryo, primary sex ratios are variable, have a heritable genetic basis, and do not appear to be affected by temperature. Our results suggest that the variable sex ratios could be under the control of multiple loci, and future work should focus on understanding the loci involved and the mechanism by which sex determination occurs in sciarids. More generally, this system offers excellent opportunities to explore polygenic control of sex determination, as well as the interplay between zygotic, maternal and environmental contributions to sex determination.

## Supplementary information

Supplementary Figure 1

## Data Availability

Data produced in this study and code used in analyses are available in the following GitHub repository: https://github.com/RossLab/Lycoriella_sex_ratios.
