# Supplementary material for: Maternal inheritance of primary sex ratios in the dark-winged fungus gnat Lycoriella ingenua
Source: Heredity (Edinb). 2026 Jan 29;135(2):113–9. doi: 10.1038/s41437-026-00821-0 (PMC12891496; doi:10.1038/s41437-026-00821-0)
Supplement: Supplementary file 1 — Supplementary Figure 1 [file 41437_2026_821_MOESM1_ESM.docx]

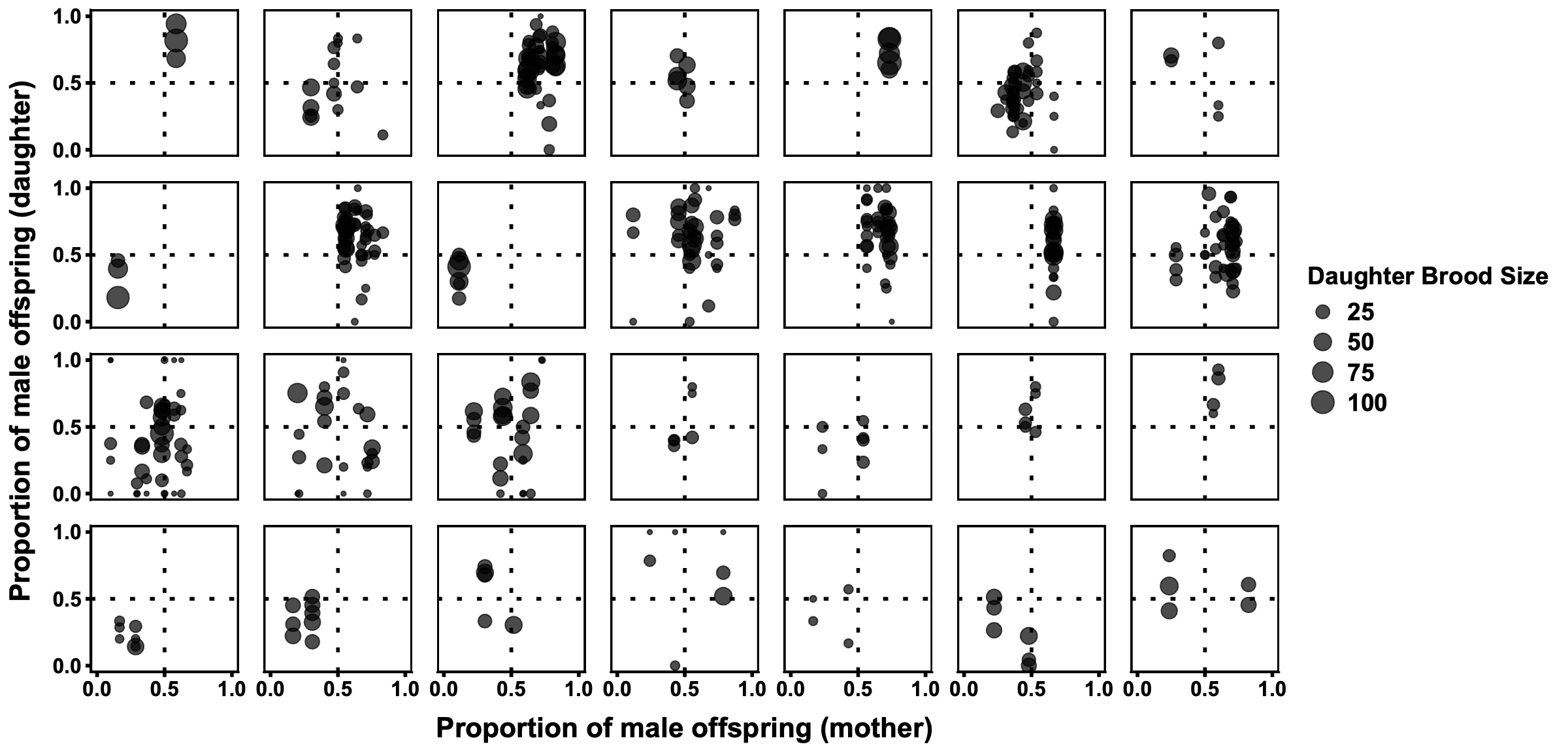


**Supplementary Figure 1.** Mother versus daughter primary sex ratios, separated by isofemale line (i.e. all broods within a plot are derived from the same founding female).
